# Practice of Oral and Maxillofacial Surgery in Nepal: Its Scope and Influencing Factor

**DOI:** 10.1155/2019/2891708

**Published:** 2019-03-24

**Authors:** Anjani Kumar Yadav, Ashok Dongol, Pradeep Acharya, Mehul R. Jaisani

**Affiliations:** Department of Oral and Maxillofacial Surgery, B. P. Koirala Institute of Health Sciences, Dharan, Nepal

## Abstract

**Objectives:**

Oral and maxillofacial surgery is a relatively newer and growing specialty of dentistry in Nepal whose scope is not yet estimated. The objective of this study was to estimate the scope and the factors influencing the scope of oral and maxillofacial surgery in Nepal.

**Study Design:**

In this cross-sectional study, all the oral and maxillofacial surgeons who were registered in the Nepalese Association of Oral and Maxillofacial Surgeons (NAOMS) were included (purposive sampling). The structured questionnaires were distributed to them, the collected data were entered in Microsoft excel 2010, and variables were analyzed using descriptive statistics (percentage) by SPSS 16.0.

**Results:**

Out of 46 questionnaires distributed, 35 were responded and returned (response rate = 76%). Majority of participants (77.1%) were in 30–39 years of age group. Male to female ratio was 4 : 1. More than half (68.6%) of the participants had practice experience of <5 years, and none had practice experience of >20 years. The ratio of the surgeons practiced in Medical/Dental Teaching Hospital to those in Government Hospital was 3 : 1. Sixteen (45.7%) participants practiced in Capital Valley and none in Far-Western Development Region. Traumatology was practiced by thirty-four (97.1%) participants although only twenty-three (65.7%) participants had primary interest in it. Each of oncology, orthognathic surgery, implantology, and cleft lip/palate surgery was performed by <8% of the participants. Common factors influencing the practice were inadequate training (71.4%) and insufficient facilities/infrastructures (45.7%).

**Conclusion:**

The scope of oral and maxillofacial surgery is limited in Nepal, and oncology, implantology, cleft lip/palate, and orthognathic surgery have received little attention.

## 1. Introduction

Oral and maxillofacial surgery (OMFS) is the specialty of dentistry which is recently introduced in Nepal and is still in the growing phase. Initially, this specialty was involved in management of pathologies associated with the oral cavity and jaw such as the cyst and tumor, but now the scope has expanded to maxillofacial trauma, cleft lip and palate surgery, head and neck oncology, salivary gland diseases, and temporomandibular joint disorders. People in today's world are more health conscious and are aware of the different medical specialties, and they prefer to visit the specialists for any kind of health-related problems. Besides that the oral and maxillofacial surgeons attend to a large number of primary patients, they also receive referrals from dental and medical professionals and also from emergency services. Oral and maxillofacial surgery is the only specialty in dentistry which is closely associated with other medical departments. The results of a study conducted in England in the Department of Oral and Facial Surgery, Sunderland District General Hospital, showed that most medical and dental practitioners had heard of the specialty of OMFS but lacked information about its full scope [1]. Conversely, 79% of the general public had not heard of OMFS, and 74% of the general public did not comprehend the role of OMFSs. Similarly, the scope of practice and the factors influencing the pattern of practice in Nepal have not been estimated yet. Therefore, this study was aimed to determine the scope of practice and factors influencing the pattern of services in different geographical regions of Nepal to gather information which will be utilized for organization of training and health care planning.

## 2. Materials and Methods

We designed a cross-sectional and questionnaire-based study on humans. The participants of this study were oral and maxillofacial surgeons of Nepal. All the oral and maxillofacial surgeons registered in Nepalese Association of Oral and Maxillofacial Surgeons were included in this study (purposive sampling), and the expected sample size was 46. A structured questionnaire was distributed to them by hand. The parameters measured were age, sex, duration of practice, subspecialty of interest, and geographical location of practice and factors influencing the choice of practice. The collected data were entered in Microsoft excel 2010 and analyzed using SPSS 16.0, and descriptive statistic like percentage was calculated. Permission was taken from “Institutional Review Committee” before commencing the study. Procedures of the research were explained to the participants, and informed written consent was obtained from each participant.

## 3. Results

Out of 46 participants, thirty-five (76%) responded the questionnaires. The number of male participants was twenty-eight (80%) and that of female participants was only seven (20%). The age distribution of the participants showed that majority of participants (*n*=22, 62.9%) were aged 30–39 years while there was no participants of age 60 years or above (Table 1). Majority of the participants (*n*=24, 68.6%) had practiced for less than 5 years, and there was no participant with practice experience of 20 years or more (Table 2).

**Figure 1:**
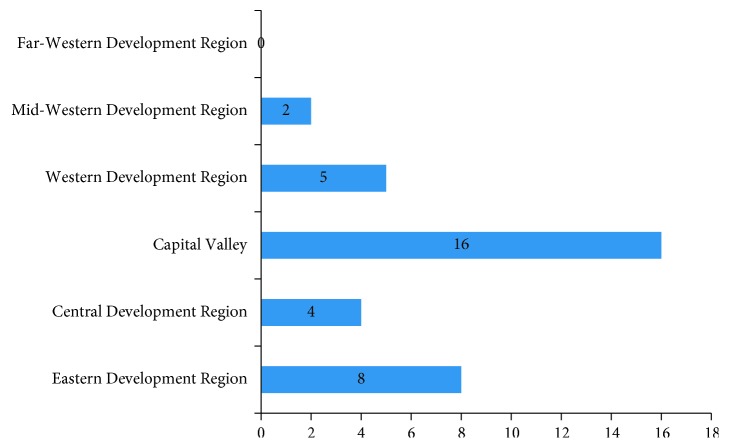
Geographical distribution of practice of oral and maxillofacial surgeons in Nepal.

**Figure 2:**
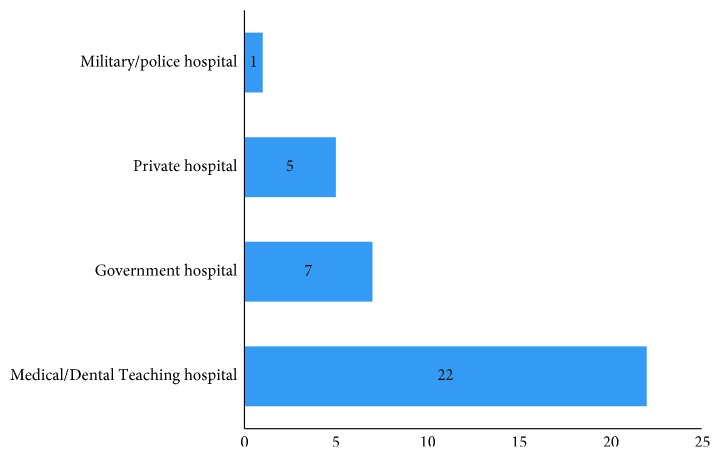
Setting of practice of oral and maxillofacial surgeons in Nepal.

The geographical distribution showed that most of the surgeons (*n*=16, 45.7%) had practiced in Capital Valley (Kathmandu, Bhaktapur, and Lalitpur) and none in Far-Western Development Region (Figure 1). Similarly, majority of the surgeons (*n*=22, 62.9%) had practiced in Medical/Dental Teaching Hospital, and none was involved only in clinical practice (Figure 2).

Thirty-four out of thirty-five participants (97.1%) were involved in traumatology. Oncology, orthognathic surgery, implantology, and cleft lip and palate surgery were practiced by less than 8% of the respondents (Table 3).

Twenty-three (65.7%) of the participants had primary interest in traumatology, and 17 (49.6%) participants had primary interest in orthognathic surgery and TMJ surgery each. Less than 10% of the participants had primary interest in dentoalveolar surgery, infection and microbiology, implantology, and pathology (Table 4).

The choice of area of interest was mainly affected by a lack of training/exposure (*n*=25, 71.4%). Financial reward and research focus had the least influence (*n*=6, 17.1% each) (Table 5).

Open reduction and internal fixation (ORIF) was the method used by the highest percentage of the participants (*n*=34, 97.1%) to treat maxillofacial fracture, while none of the participants used suspension wiring (Table 6). Among those who perform ORIF, only 85.7% (*n*=30) used plates and screws routinely.

The main confrontation to the cleft lip and palate surgery indicated by most surgeons who were not involved in cleft surgery (*n*=30) was limited training (*n*=17, 56.7%), followed by most patients being treated free of cost by charitable projects (*n*=15, 50%) (Table 7).

Among those who were not involved in orthognathic surgery (*n*=30), the main reason given for noninvolvement was limited training (*n*=23, 76.7%) (Table 8). Similarly, the main reason given for noninvolvement in oncology (*n*=28) was inadequate backup/support (*n*=22, 78.6%) and limited training (*n*=14, 50%), followed by poor facility (*n*=13, 46.4%) (Table 9). Among those who were involved in oncology (*n*=7), four participants routinely performed reconstruction of surgical defects and three participants did not.

The reasons given for noninvolvement by the participants who were not involved in TMJ surgery (*n*=21) were limited training (*n*=17, 80.9%), inadequate backup/support (*n*=7, 33.3%), poor facility (*n*=5, 23.8%), and patient lack of motivation (*n*=3, 14.3%).

## 4. Discussion

Oral and maxillofacial surgery is the subspecialty of dental/medical science that deals with the management of varieties of pathologic conditions of the jaw, mouth, and face. However, it has confrontation of low levels of awareness amongst the public and other medical/paramedical professionals [2–5]. Therefore, to the best of our knowledge, this research could be the first effort in estimating the current level of practice and the factors influencing the development of this specialty in Nepal. Although this study was conducted among the oral and maxillofacial surgeons of Nepal who were registered in Nepalese Association of Oral and Maxillofacial Surgeons (NAOMS) despite the fact that there were few surgeons who were not registered in the association, 76% response rate of the questionnaires, as we assume, could represent the status of the whole oral and maxillofacial surgeons of Nepal. There is a similar study conducted in Nigeria which shows the response rate of only 56% [6], as compared to 76% response rate in this study. This difference would indicate that most of the oral and maxillofacial surgeons of Nepal are interested to know the level of practice and factors influencing the practice in Nepal.

This study revealed the predominance of male surgeons (80%) that is consistent with the findings of male dominance in surgical specialties in studies conducted by Brennan et al. [7]. In contrast to this study, our study had higher percentage of female (20%) which indicates the increase in interest of female towards surgical specialties [8]. The greater proportion of surgeons in this study was in the 30–39 years age group (77.1%) which is less than that reported in similar studies in Australia [7] and Nigeria [6]. Similarly, 94.3% of the surgeons in this study have been in practice for ≤10 years, and only 5.7% were in practice for >10 years, which is quite different from a similar study in Nigeria [6] where 50% of surgeons were in practice for ≤10 years and 50% were in practice for >10 years. This difference in the age group and years in practice would show that oral and maxillofacial surgery in Nepal has been recently evolved and is still in the growing phase. In this study, the practice of oral and maxillofacial surgery is centered in capital valley (45.7%) which is could be due to more job opportunity with good facilities/infrastructure in that region. This study had no participant working in the Far-Western Development Region which indicates that either the scope of oral and maxillofacial surgery is limited or there is lack of facilities/infrastructure in that region. In the present study, majority of the surgeons (62.9%) practiced in Medical/Dental Teaching Hospital and only 20% surgeons practiced in Government Hospital which is quite different from a study conducted in Nigeria [6]. This difference in practice reflects the government policy in Nepal, which either does not encourage the surgeons to work in Government Hospital or there is lack of job opportunities or infrastructure in Government Hospital.

Traumatology, both soft tissue injury and maxillofacial bone fracture, is the subspecialty of maxillofacial surgery more commonly practiced by maxillofacial surgeons in Nepal [9, 10], which is similar with findings from a study conducted by Hofman et al. [11]. Open reduction and internal fixation with plates and screws is the choice of method for treatment of maxillofacial fracture, as reported in this study. This differs from a similar study in Nigeria [6] where only 53% of surgeons routinely perform open reduction with plates and screws which could be due to either good infrastructure for trauma in Nepal, or Nepalese patients can better afford the cost of miniplates. Although 45.7% of the participants in this study had primary interest in oncology, only 20% were involved and the main challenge to oncological maxillofacial surgery observed in this study is lack of training and backup/support. This is different from the finding of similar study in Nigeria where the challenge facing oncological maxillofacial surgery was late patients' presentation [6]. This difference suggests that Nepalese patients seek oncological treatment earlier, but the surgeons in Nepal lack competency in oncological maxillofacial surgery. Although 20% of the participants practiced in Government Hospital, none of them are involved in cleft lip and palate surgery and those who are involved in cleft lip and palate surgery, all practice in private hospitals. This shows the government policy which does not promote cleft surgery in government hospitals.

In this study, only 65.7% of the participants had primary interest in traumatology, whereas 97.1% have been practicing traumatology. Similarly, almost 50% of the participants had primary interest in oncology, temporomandibular joint surgery, and orthognathic surgery, whereas <8% have been practicing these subspecialties. This difference in subspecialties of interest and subspecialties being practiced could be due to lack of adequate training, poor facilities, and inadequate backup/support.

## 5. Conclusion

This study has delineated the present pattern of practice and the factors influencing the practice of oral and maxillofacial surgery in Nepal. This study is the first effort done to evaluate the pattern of practice and factors influencing the services provided by oral and maxillofacial surgeons in Nepal. The lack of adequate training as the major limiting factor for oncology, implantology, cleft lip and palate surgery, and orthognathic surgery necessitates that younger generation surgeons require structured training in these subspecialties. The capital valley focused practice of oral and maxillofacial surgery drew an attention towards the distribution of surgeons, which need to be addressed to provide these specialized services to the population throughout the country. Although the sample size is small, this study will provide the baseline data, and further study with large sample size can be planned in future to observe the changes in pattern of practice over the time.

## Figures and Tables

**Table 1:** Age distribution of oral and maxillofacial surgeons in Nepal.

Age range (years)	Frequency (*n*)
<30	6 (17.1%)
30–39	27 (77.1%)
40–49	1 (2.9%)
50–59	1 (2.9%)
Total	35 (100%)

*n* = number of participants.

**Table 2:** Practice experience of oral and maxillofacial surgeons in Nepal.

Years in practice	Frequency (*n*)
≤5	24 (68.6%)
6–10	9 (25.7%)
11–15	0 (0%)
16–20	2 (5.7%)
≥20	0 (0%)
Total	35 (100%)

*n* = number of participants.

**Table 3:** Subspecialties of oral and maxillofacial surgery being practiced in Nepal.

Subspecialties practiced	Number of participants
Traumatology	34 (97.1%)
Oncology	7 (20%)
Dentoalveolar surgery	27 (77.1%)
Infection and microbiology	28 (80%)
Orthognathic surgery	5 (17.3%)
Implantology	5 (17.3%)
TMJ surgery	14 (40%)
Cleft lip and palate surgery	5 (17.3%)
Pathology	29 (82.9%)

**Table 4:** Subspecialties of interest of oral and maxillofacial surgeons of Nepal.

Subspecialties of interest	Number of participants
Traumatology	23 (65.7%)
Oncology	16 (45.7%)
Dentoalveolar surgery	6 (17.1%)
Infection and microbiology	3 (8.6%)
Orthognathic surgery	17 (49.6%)
Implantology	5 (17.3%)
TMJ surgery	17 (49.6%)
Cleft lip and palate surgery	10 (28.6%)
Pathology	8 (22.9%)

**Table 5:** Factors influencing choice/area of interest of subspecialties of oral and maxillofacial surgery in Nepal.

Factors influencing choice/area of interest	Number of participants
Training/exposure	25 (71.4%)
Availabilities of facilities	16 (45.7%)
Sheer interest/flare	10 (28.6%)
Financial reward	6 (17.1%)
Research focus	6 (17.1%)

**Table 6:** Method of treating maxillofacial fracture in Nepal.

Methods	Number of participants
Wiring	5 (14.3%)
Maxillomandibular fixation	12 (34.3%)
Open reduction and internal fixation	34 (97.1)
Splint	5 (14.3%)

**Table 7:** Reasons of noninvolvement by respondents not involved in the cleft lip and palate surgery (*n*=30).

Reasons	Number of participants
Poor facility	4 (13.3%)
Limited training	17 (56.7%)
Inadequate backup/support	9 (30%)
Patient lack of motivation	1 (3.3%)
Most patient being treated free of cost by charitable projects	15 (50%)

*n* = total number of participants not involved in the cleft lip and palate surgery.

**Table 8:** Reasons for noninvolvement in orthognathic surgery (*n*=30).

Reasons	Number of participants
Limited training	23 (76.7%)
Inadequate backup/support	12 (40%)
Patient lack of motivation	10 (33.3%)
Poor facilities	8 (26.7%)

**Table 9:** Reasons for noninvolvement in oncology (*n*=28).

Reasons	Number of participants
Limited training	14 (50%)
Inadequate backup/support	22 9 (78.6%)
Late presentation of the patient	8 (28.6%)
Poor facilities	13 (46.4%)
Less financial reward	1 (3.6%)

## Data Availability

The data used to support the findings of this study have not been made available to secure the privacy right of the participants and to prevent duplication of the research by manipulating the data. If needed during the review process, it can be provided to the editor upon request.
